# Role and Diagnostic Performance of Host Epigenome in Respiratory Morbidity after RSV Infection: The EPIRESVi Study

**DOI:** 10.3389/fimmu.2022.875691

**Published:** 2022-05-10

**Authors:** Sara Pischedda, Irene Rivero-Calle, Alberto Gómez-Carballa, Miriam Cebey-López, Ruth Barral-Arca, Jose Gómez-Rial, Jacobo Pardo-Seco, María-José Curras-Tuala, Sandra Viz-Lasheras, Xabier Bello, Ana B. Crujeiras, Angel Diaz-Lagares, María Teresa González-López, Federico Martinón-Torres, Antonio Salas, GENDRES consortium

**Affiliations:** ^1^ Genetics, Vaccines, Infectious Diseases and Pediatrics Research Group (GENVIP), Instituto de Investigación Sanitaria de Santiago, Santiago de Compostela, Santiago de Compostela, Spain; ^2^ Translational Pediatrics and Infectious Diseases, Department of Pediatrics, Hospital Clínico Universitario de Santiago de Compostela, Santiago de Compostela, Spain; ^3^ GenPoB Research Group, Instituto de Investigacinó Sanitaria (IDIS), Hospital Clínico Universitario de Santiago (SERGAS), Unidade de Xenética, Santiago de Compostela, Spain; ^4^ Centro de Investigación Biomédica en Red de Enfermedades Respiratorias (CIBER-ES), Madrid, Spain; ^5^ Epigenomics in Endocrinology and Nutrition Group, Epigenomics Unit, Instituto De Investigación Sanitaria De Santiago De Compostela (IDIS), Complejo Hospitalario Universitario De Santiago De Compostela (CHUS/SERGAS), Santiago de Compostela, Spain; ^6^ Centro De Investigación Biomédica En Red Fisiopatología De La Obesidad Y Nutrición (Ciberobn), Madrid, Spain; ^7^ Cancer Epigenomics, Epigenomics Unit, Translational Medical Oncology (Oncomet), Instituto De Investigación Sanitaria De Santiago De Compostela (IDIS), Complejo Hospitalario Universitario De Santiago De Compostela (CHUS/SERGAS), Santiago De Compostela, Spain; ^8^ Centro De Investigación Biomédica En Red Cancer (CIBERONC), Madrid, Spain; ^9^ Complejo Hospitalario Universitario de Ourense (SERGAS), Ourense, Spain; ^10^ Instituto de Ciencias Forenses, Facultade de Medicina, Universidade de Santiago de Compostela, Santiago de Compostela, Spain

**Keywords:** RSV, DNA methylation, immune system, respiratory sequelae, recurrent wheezing, asthma

## Abstract

**Background:**

Respiratory syncytial virus (RSV) infection has been associated with the subsequent development of recurrent wheezing and asthma, although the mechanisms involved are still unknown. We investigate the role of epigenetics in the respiratory morbidity after infection by comparing methylation patterns from children who develop recurrent wheezing (RW-RSV), subsequent asthma (AS-RVS), and those experiencing complete recovery (CR-RSV).

**Methods:**

Prospective, observational study of infants aged < 2 years with RSV respiratory infection admitted to hospital and followed-up after discharge for at least three years. According to their clinical course, patients were categorized into subgroups: RW-RSV (*n* = 36), AS-RSV (*n* = 9), and CR-RSV (*n* = 32). The DNA genome-wide methylation pattern was analyzed in whole blood samples, collected during the acute phase of the infection, using the Illumina Infinium Methylation EPIC BeadChip (850K CpG sites). Differences in methylation were determined through a linear regression model adjusted for age, gender and cell composition.

**Results:**

Patients who developed respiratory sequelae showed a statistically significant higher proportion of NK and CD8T cells (inferred through a deconvolution approach) than those with complete recovery. We identified 5,097 significant differentially methylated positions (DMPs) when comparing RW-RSV and AS-RVS together against CR-RSV. Methylation profiles affect several genes involved in airway inflammation processes. The most significant DMPs were found to be hypomethylated in cases and therefore generally leading to overexpression of affected genes. The lead CpG position (cg24509398) falls at the gene body of *EYA3* (*P*-value = 2.77×10^-10^), a tyrosine phosphatase connected with pulmonary vascular remodeling, a key process in the asthma pathology. Logistic regression analysis resulted in a diagnostic epigenetic signature of 3-DMPs (involving genes *ZNF2698*, *LOC102723354* and *RPL15*/*NKIRAS1*) that allows to efficiently differentiate sequelae cases from CR-RSV patients (AUC = 1.00). Enrichment pathway analysis reveals the role of the cell cycle checkpoint (FDR *P*-value = 4.71×10^-2^), DNA damage (FD*P*-value = 2.53×10^-2^), and DNA integrity checkpoint (FDR *P*-value = 2.56×10^-2^) in differentiating sequelae from CR-RSV patients.

**Conclusions:**

Epigenetic mechanisms might play a fundamental role in the long-term sequelae after RSV infection, contributing to explain the different phenotypes observed.

## Introduction

Respiratory syncytial virus (RSV) is a common pathogen that infects children by two years of age and is the leading global cause of hospitalization of infants (1). It is the principal cause of acute lower respiratory infections (ALRI) in young children, and it is associated with morbidity and mortality in childhood (2). Approximately 34 million new ALRI episodes in children worldwide were attributable to RSV (1), a huge number that resulted in 3.2 million hospital admissions and almost 60,000 global childhood deaths each year. No effective vaccines are available to treat RSV yet, although some good candidates are being tested in large-state clinical trials (3, 4). For the time being, palivizumab is the only monoclonal antibody approved to prevent severe RSV in infants and children at high risk for severe disease (5, 6).

Besides the acute burden of RSV, a growing body of evidence from epidemiology data supports that RSV infection in the first three years of life can be directly correlated with long-term respiratory morbidities, such as recurrent wheezing and asthma (7, 8). It has been observed that RSV, like other respiratory viruses, causes a “*hit and run*” phenomenon, characterized by the increased risk of developing recurrent wheezing and asthma in childhood after the infection, as a permanent phenotype that persists long after the virus clearance (9). Wheezing is the typical high-pitched, whistling sound made during breathing. Asthma, on the other hand, is characterized by abnormalities in lung function that include variable airway obstruction and increased bronchial reactivity (10). Recognizing asthma is usually obvious and most of the time asthmatic patients also report wheezing episodes; however, it is very challenging to predict and distinguish which children will present only early-life symptoms, from those whose symptoms persist, and those who may develop definitive wheeze or asthma (11).

The risk of wheezing and/or asthma incidence has been increasingly related to a combination of genetic and environmental factors as well as the severity of the respiratory infection (12–14). Several efforts have been made to establish the ultimate causes underlying the apparition of respiratory sequelae as a consequence of RSV infection, but further complementary studies are still necessary to understand the underlying genetic and molecular mechanisms. It has been hypothesized that prenatal interaction between the maternal and the child immune system as well as the high cytokines production after the infection can affect the lung structure and function inducing changes in the regulation of immune response (15).

The immune system plays a pivotal role in the sequelae reported in patients suffering from respiratory infections. The immune response shows many functional differences in neonates and adults, and these differences might be associated with epigenetic modifications of genes that control inflammation and immune response (16). Thus, the modulation of epigenetic mechanisms governs the immune cell phenotype and function allowing the external environment to influence the immune response outcome (17).

Epigenetic regulation behaves as a dynamic interface between genome and environment; in the case of viral infection, this regulation in host defense cells is directly related to disease development (18). Pieces of evidence support the involvement of epigenetic mechanisms in the modulation of the interaction between host and pathogen (19), as it also occurs as a consequence of vaccination [e.g. rotavirus infection (20)]. For instance, it has been shown that some viral infections (e.g. by herpesvirus, KSHV, EBV) can lead to the modification of host epigenetic marks, and this probably contributes to the establishment of latency and some pathogenic roles (21, 22).

DNA methylation involves the addition of methyl groups to the DNA molecule and is the major epigenetic factor influencing gene activities. It is generally associated with a decrease of gene expression when it occurs in promoters; however, according to recent evidence (23, 24) the effect of DNA methylation occurring in low CpG density regions, such as the gene body, or intergenic regions, might have also great impact on the regulation of gene expression, with repressive or permissive effects. Recently, Elgislouli and colleagues (25) observed an alteration of the methylation pattern of a particular enhancer region in the perforin 1 gene (*PRF1*), an essential cytotoxic protein for the control of viral infection, occurring after severe RSV infection. Infants hospitalized with exacerbated RSV-induced bronchiolitis exhibited decreased methylation of the perforin-1 enhancer after four years of follow-up, suggesting that immune response changes due to RSV could persist even years after infection (26). Besides, Wang et al. (18) showed that cultured bronchial human epithelial cells (BECs) infected with RSV presented a high expression level of the gene *NODAL* (a member of the TGF-beta superfamily), whose promoter was found to be hypermethylated in normal BECs, resulting in increased Th2 and Th17 skewing of T cells. In murine models, it was observed an overexpression of the demethylase genes *Kdm5b* and *H3K4*, in dendritic cells after RSV infection (27); expression of these genes has the potential to repress transcription of type I IFN and other innate cytokines, causing a decrease of pro-inflammatory cytokines and expression of a Th2 phenotype. Methylation of *H3K4* in regulatory T cells by the histone methyltransferase SMYD3 is necessary to control inflammation in the lungs after RSV infection (28).

The aim of the present study is to analyze the DNA methylation changes related to the development of wheezing and/or asthma induced after RSV infection. To the best of our knowledge, this is the first time that an epigenomic approach is applied to the investigation of epigenetic modifications due to RSV infection and the subsequent long-term respiratory sequelae.

## Material and Methods

### Study Subjects and Design

EPIRSVI is a prospective, transversal, observational study of children admitted to the Hospital Clínico Universitario de Santiago de Compostela, and the Complejo Hospitalario Universitario de Ourense, part of the GENDRES consortium (www.gendres.org), for a respiratory infection due to RSV (**Supplementary Table 1**). The recruitment of patients was performed from 2010 to 2015, during their acute phase of RSV infection (within seven days from the beginning of symptoms). Patients, independently from their age, were all followed up from their recruitment for a time-lapse of at least three years after discharge to specifically monitor the onset of wheezing or asthma. Patients were categorized according to their clinical course into three different subgroups (*n* = 77): (a) recurrent wheezing RSV cases (RW-RSV; *n* = 36), (b) asthma RSV cases (AS-RSV; *n* = 9); and (c) not-wheezing/asthma RSV cases with complete recovery (CR-RSV; *n* = 32). The three groups matched perfectly for age, gender, and disease severity at the time of sample collection.

Wheezing was determined here as a respiratory episode occurring with wheezing lasting more than one day. The interval between two episodes was defined as a period of at least seven days without respiratory symptoms. Instead, recurrent wheeze was defined as three or more episodes of wheezing during the first year of life. The diagnosis of asthma in preschool children was carried out by a pediatric pulmonologist based on the reiterated presence of compatible symptoms (several episodes of bronchial obstruction, generally witnessed by the physician), adequate response to regular treatment, and the exclusion of other alternative diagnoses, following the international practice guidelines. From the clinical perspective, the diagnosis of asthma in these children involves a series of peculiarities inherent to patients of this age, such as limitations for performing pulmonary function tests, greater attention to differential diagnoses, limited response to common asthma treatments, and a high probability of symptoms remission during childhood.

### DNA Isolation and Methylation Profiling

Blood samples were collected in EDTA tubes, within 24/48 hours from the hospital admission of each patient. Genomic DNA was isolated using the Wizard^®^ Genomic DNA Purification Kit (PROMEGA) QIAamp DNA mini kit (Qiagen) according to the manufacturer’s instructions. After DNA extraction, DNA was purified, and concentration was evaluated using a Nanodrop and Picogreen assay.

Genomic DNA from whole blood samples was bisulfite-treated using the EZ-96 DNA Methylation kit (Zymo Research Corp) following the manufacturer’s recommendations for Infinium assays. The technique consists of the conversion of unmethylated cytosine residues to uracil through deamination, while leaving the methylated cytosine (5-mC) unaffected.

Following bisulfite conversion, the samples were hybridized to Illumina Infinium MethylationEPIC BeadChip. This microarray allows the examination of >850K methylation sites quantitatively across the genome at single-nucleotide resolution. The microarray coverage includes 99% of the RefSeq genes, 95% of CpG islands, non-CpG islands, and differentially methylated sites, high coverage of transcription factors binding sites, miRNA promoter regions, and enhancer sequences. We compared the methylome of RW-RSV, AS-RSV, with CR-RSV to evaluate whether the divergent epigenetic marks could be specific for the development of recurrent wheezing and/or asthma.

### Data Processing, Cell Deconvolution and Statistical Analysis

DNA methylation quality control, processing, normalization, and statistical analyses were performed in the statistical R software (29), using different Bioconductor packages, and following the workflow of EPIC methylation analysis described recently by Fortin et al. (30). The raw intensity files (IDAT), were imported into R and were preprocessed and transformed into *β* and M-values using the *minfi* (v1.30.0) package (31, 32). The *β*-values are defined as the intensity ratio of the methylated signals over the total (methylated and unmethylated) signals for each site. Their values range between 0, illustrating that the position is not methylated at all in all the cells and 1, indicating that all cells in the sample have a given position methylated (33). The M-values were calculated as the log_2_ ratio of the intensities of methylated probes *vs.* unmethylated probes; their values range from –1 to 1, where values close to 0 define a similar intensity between the methylated and unmethylated probes, positive M-values indicate that there are more methylated than unmethylated molecules, while negative M-values mean the opposite (34).

Counting blood cells is a necessary step to adjust for individual differences in cellular heterogeneity in the blood sample from which genomic DNA was extracted. Because of the extreme cell-type specificity of DNA methylation (35), variations in cell-type composition between phenotypes can confound analyses and bring to a wrong interpretation of the results. Therefore, before checking for differentially methylated positions (DMPs), we used the package *FlowSorted.Blood*.*EPIC* and its modified function *estimateCellCounts2* for the estimation of blood cell type composition. At the base of the function, there is a modification of the Houseman algorithm (36), that allows estimating the relative proportions of white blood cell subtypes, namely, CD4+ T-lymphocytes, CD8+ T-lymphocytes, natural killer (NK) cells, B-lymphocytes, monocytes, and neutrophils.

Infinium 850K has two kinds of probes, Infinium I and Infinium II, that are not directly comparable, therefore, a correction must be applied to control for design bias. The normalization procedures were performed using the function *preprocessQuantile* available in the *minfi* package (37). Moreover, probes underwent several filtering processes, by removing probes with *P*-value > 0.01 and probes located in the sex chromosomes. Additionally, sites containing SNPs or with a minor allele frequency (MAF) < 0.05 were also excluded from the data analysis because probe binding might be affected by genetic variation in the binding area. Finally, we removed probes known to have cross-reaction.

DMPs were identified with *limma* (38) assuming a linear model, where M-values of each probe were used as quantitative dependent variables in all analyses, and including cell composition, age, and gender as covariates in all the models. After running the linear model, we applied the statistical analysis using an empirical Bayes method to moderate standard errors. DMPs were filtered using a significance threshold of adjusted *P*-value < 0.01 [Benjamini–Hochberg method (39)].

We used a Principal Component Analysis (PCA) to compare DNA methylation of children with complete recovery (CR-RVS) and those with respiratory sequelae after an RSV infection (RW-RVS + AS-RVS). The threshold Delta *β* of 0.1 and adjusted *P*-value < 0.01 were used to identify candidate DMPs among the comparison groups. The diagnostic efficiency of the most significant candidate DMPs was evaluated using receiver operating characteristic (ROC) curve analyses. Furthermore, the candidate DMPs were included in the logistic regression analysis using the Parallel Regularised Regression Model Search (PReMS) (40) method that allows identifying the minimum epigenetic signature with the highest diagnostic performance to differentiate between RW/AS-RSV and CR-RSV groups. This logistic regression model balances small positions number with accurate discrimination, minimizing the number of biomarkers selected in the signatures. The optimal model size (with the lowest out-of-sample log-likelihood) was determined using 20 cross-validation folds. In addition to the best model, results for model sizes with predictive log-likelihood within one-standard error of the best model is also shown. We randomly split the cohort into 75% training and 25% test sets, ensuring equal proportions of RW/AS-RSV and CR-RSV in each set. The epigenetic signature was identified in the training set and afterwards validated in the test set. Wilcoxon test was used to assess statistical significance between patient groups.

To deeply analyze the functional interpretation of our reported differential probes, we performed pathways enrichment analysis on the candidate DMPs applying the *methylglm* and *methylRRA* functions of the R *methylGSA* package (41). To adjust for the number of CpGs, the first method employs a logistic regression model incorporating the number of CpGs as a covariate, while the second approach makes use of a Robust Rank aggregation (RRA) procedure. Indeed, the number of CpGs is variable for genes of similar length and DNA methylation studies give rise to multiple CpG association *P*-values per gene.

To find co-methylated modules related to respiratory sequelae, we built a signed weighted correlation network using the *WGCNA* package (42) with the CpGs showing the most different *β*-values between samples (top 25% with a higher variance; *n* = 123,617). We selected a soft-thresholding power of 12 based on the criterion of scale-free topology after testing a set of candidate powers (**Supplementary Figure 1A**). As module detection parameters, we chose a minimum module size of 30, a medium sensitivity for cluster splitting and a 0.25 as dendrogram cut heigh threshold for module merging. We calculated gene significance (GS) to detect significant associations between modules/genes and phenotype. Module membership (MM), as a measurement of intramodular connectivity, was also calculated by correlating the methylation profile with the eigengene of a given module. We explored the correlation between GS and MM and calculated the average absolute gene significance for all genes within a module to find the most important modules. The top hub genes within the most relevant module were selected using both MM > 0.8 and GS ≤ –0.7 as thresholds. We studied the biological significance of the most important module related to the trait by performing an over-representation analysis through the *Clusterprofiler* R package (43), and using Gene Ontology (GO) database as a reference.

## Results

### Patient Characteristics

**Table 1** summarizes the clinical characteristics of the subjects studied and maintained for the downstream statistical analysis after filtering raw data (*n* = 68), and it provides information regarding the most important risk factors related to the development of asthma in early childhood. For the clinical characteristics of the full cohort see **Supplementary Table 1**. More males than females represented sequelae and complete recovery groups (60.7% and 70.0% for CR-RSV and RW/AS-RSV, respectively). Concerning disease severity, all children were hospitalized because of RSV infection and their admission lasted > 5 days. Most subjects were diagnosed with bronchiolitis and required oxygen during their hospital admission. Most children were under six months of age; however, a significant difference (*P* < 0.001) was observed in the mean age of both groups, being children with respiratory sequelae slightly younger than patients with complete recovery after RSV. No comorbidities were present in the study cohort apart from prematurity (see **Table 1**). Thus, admissions prior to RSV were due to prematurity, bronchiolitis (mainly), rotavirus gastroenteritis in one case and hypernatremia dehydration in another case. Most of the children admitted below the age of one year were normally more common to have oxygen requirement, suffer apneas or need of intravenous fluids due to lack of appetite, therefore the number of admissions is generally higher compared to older ages. According to the previous medical history of these patients, the only clinical variable found to be statistically significant was the personal history of atopic dermatitis (Fisher exact test; *P*-value = 0.017), while alimentary and stational allergies showed no significant difference between the two groups. The family history of asthma and respiratory problems were not found to be significantly different between groups. Finally, we observed a significant difference (Fisher exact test; *P*-value = 0.005) in the number of bronchitis episode prior to hospitalization in patients with respiratory sequelae, and a higher number of suspected bacterial infection (Fisher exact test; *P*-value <0.001) in children with normal recovery.

**Table 1:** Clinical characteristics of patients left for downstream analysis, classified as recurrent wheezing RSV (RW-RSV), asthma RSV (AS-RSV), and complete recovery RSV cases (CR-RSV).

	CR-RSV (*n* = 28)	RW/AS-RSV (*n* = 40)	*P*-value
Demographic variables			
Sex Male	17 (60.07%)	28 (70.0%)	0.447
Age in months (mean [SD])*	7.14 [5.32]	5.85[3.62]	<0.001
Ethnicity			0.184
Western Europe	24 (85.7%)	36 (90.0%)	–
Southern Europe	2 (7.1%)	1 (2.5%)	–
Southern America	1 (3.6%)	0	–
Roma	0	3 (7.5%)	–
Other	1 (3.6%)	0	–
RSV infection	28 (100.0%)	40 (100.0%)	1.000
Past medical history prior to RSV-A			
Premature	4 (14.3%)	4 (10.0%)	0.717
Atopic Dermatitis*	2 (7.1%)	13 (32.5%)	0.017
Alimentary allergies	1 (3.6%)	5 (12.5%)	0.399
Stational allergies	0	2 (5.0%)	0.508
Asthma	0	0	1.000
Admissions prior to RSV	10 (35.7%)	12 (27.0%)	0.399
Annual bronchitis prior the RSV-A*	3 (10.7%)	21 (52.5%)	0.005
Family history			
Asthma	4 (14.3%)	14 (35.0%)	0.052
Respiratory problems	5 (17.9%)	15 (37.5%)	0.053
Clinical characteristics of the RSV-H			
Respiratory distress			0.147
Mild	8 (28.6%)	4 (10.0%)	
Moderate	16 (57.1%)	29 (72.5%)	
Severe	4 (14.3%)	7 (17.5%)	
Oxygen requirement	23 (82.1%)	25 (62.5%)	0.107
Respiratory support			0.128
Non-invasive	3 (10.7%)	8 (20%)	
Mechanical	2 (7.1%)	0	
Diagnosis			0.093
Bronchiolitis	25 (89.3%)	31 (77.5%)	
Bronchospasm	0	1 (2.5%)	
Pneumonia	2 (7.1%)	2 (5.0%)	
Other	1 (3.6)	6 (15.0%)	
Bacterial superinfection suspected*	20 (71.4%)	12 (30.0%)	<0.001
Follow-up 3 years			
Hospital admission	5 (17.9%)	11 (27.5%)	0.962
Additional episodes of bronchiolitis*	7 (25%)	37 (92.5%)	<0.001

Fisher’s exact test is used to assess the association between the different variables. RSV-A, RSV admission; RSV-H, RSV hospitalization.

*Statistically significant variables.

### DMPs Between Complete Recovered and Sequelae Cases

We interrogated the genome-wide DNA methylation profiles of 77 children during their acute phase of RSV infection that experienced complete recovered or developed respiratory sequelae.

During the data preprocessing nine samples were discarded because they did not pass the initial quality control; therefore, 68 out of 77 samples were included in the statistical analysis. After removing poorly performing probes, namely, those overlapping with SNPs or hybridized to multiple locations in the genome and those located on the X and Y chromosomes, a total of 811,035 probes were preserved for downstream analysis.

**Figure 1:**
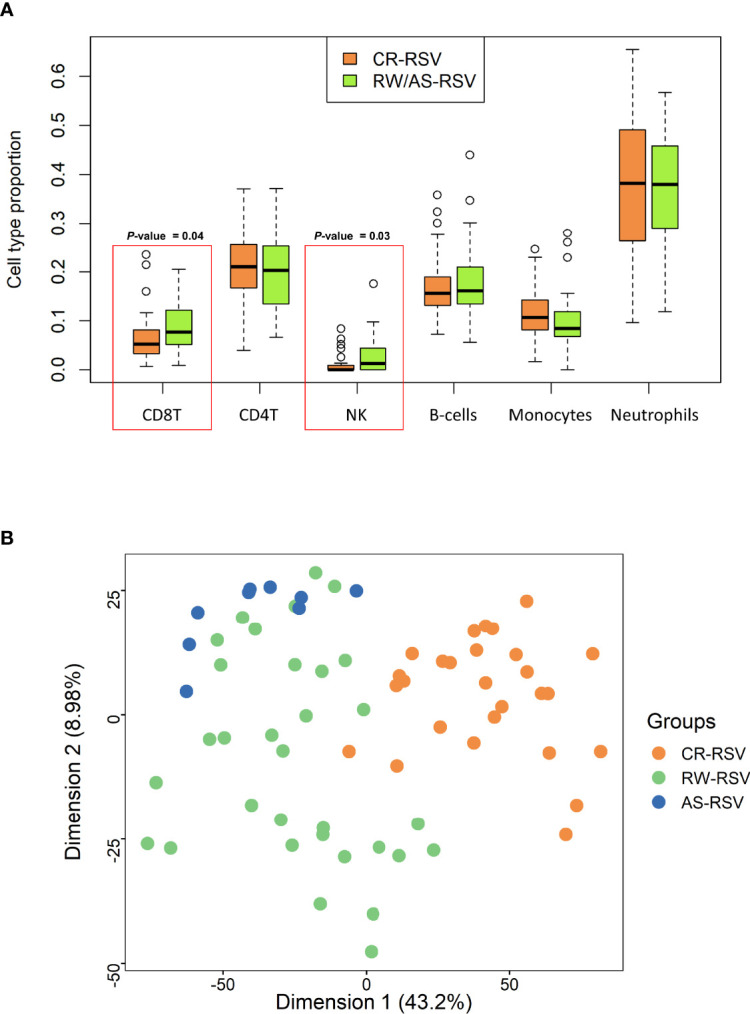
**(A)** Boxplot showing the proportion of leukocyte cell type in RW/AS-RSV and CR-RSV groups. Red rectangles highlight the two types of cells that show statistically significant differences between groups. **(B)** PCA of the significant DMPs (FDR *P*-value < 0.01) between RW/AS-RSV and CR-RSV cases.

Cell deconvolution analysis indicates a significant difference in the number of NK and CD8+T cells between sequelae and recovered cases (*P*-value = 0.03 and *P*-value = 0.04, respectively); **Figure 1A**. When we compared RW/AS-RSV against CR-RSV patients, adjusting the linear model for cell-type composition, age, and gender, we found 5,097 significantly DMPs (FDR *P*-value < 0.01), corresponding to 3,278 unique genes according to the Illumina Human Methylation EPIC manifest annotation file (44) (**Supplementary Table 2**). Among these, 1,155 DMPs were hypomethylated (22.7%) and 3,942 hypermethylated (77.3%) in CR-RSV. PCA of these significant DMPs showed a clear separation of the two groups along with the first principal component (PC1; accounting for 43.2% of the variance) (**Figure 1B**).

**Figure 2:**
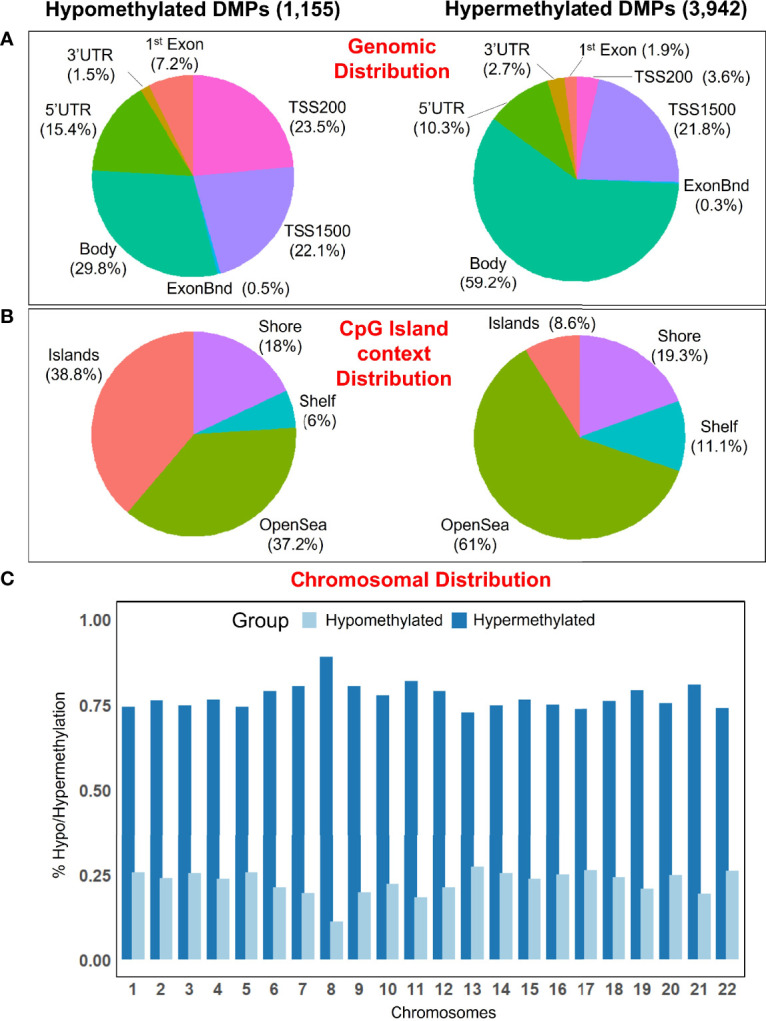
**(A)** Pie chart showing the percentage of the significant DMPs in the genomic context, and **(B)** according to their distribution in the Island context. **(C)** Barplot showing the distribution of hypomethylated and hypermethylated DMPs in the chromosomes.

There are regional differences between the distribution of statistical different hypomethylated and hypermethylated CpGs when examined in a genomic context. Thus, hypermethylated positions are more concentrated in the gene body (59.2%), while the hypomethylated positions are distributed more homogeneously along the gene body (29.8%), promoter regions (45.6%), and transcriptions start sites [TSS1500 (22.1%), TSS200 (23.5%)], with a lower amount falling at ‘UTR (15.4%) and 1^st^ Exon (7.2%) (**Figure 2A**). Island’s regions are overrepresented by hypomethylated sites (38.8%), while the OpenSea regions are more characterized by hypermethylated DMPs (61%); the distribution of DMPs at shelf and shore was quite similar between the two comparison groups (**Figure 2B**). Moreover, all chromosomes were more represented by hypermethylated DMPs than the hypomethylated ones (**Figure 2C**).

**Figure 3:**
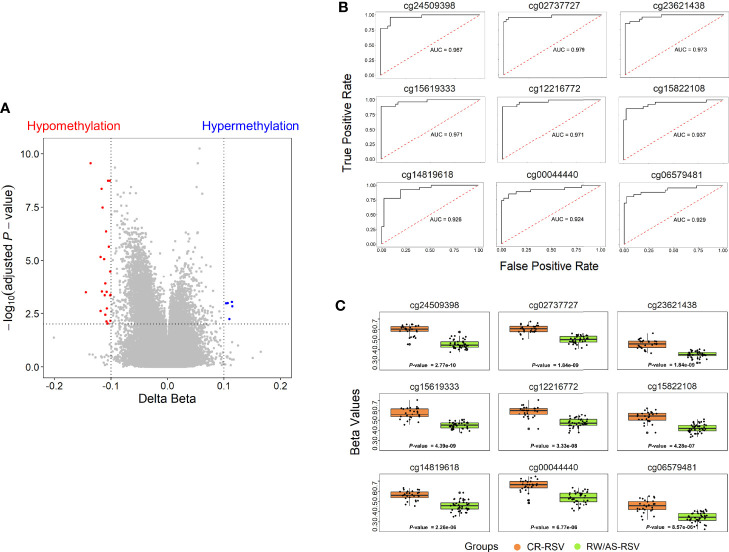
**(A)** Volcano plots for differential DNA methylation status. The x-axis shows the mean DNA methylation (*β* -value) difference, whereas the y-axis indicates the –log_10_ of the adjusted *P*-value for each CpG site. The most significant DMPs (*n* = 28) in children with respiratory sequelae when compared with children complete recovered (threshold: Delta *β* > 0.10, FDR *P*-value < 0.01) are plotted in red (hypomethylated sites) and blue (hypermethylated CpGs). **(B)** Receiver operating characteristic (ROC) curves indicate that the accuracy of the test based on the reported CpGs is very high (AUC > 90%) when comparing RW/AS-RSV and CR-RSV groups. **(C)** Boxplot of the nine most significant DMPs with a Delta *β* > 0.10 in the comparison RW/AS-RSV vs. CR-RSV cases.

To narrow down the analysis and reduce the large number of significant methylated positions observed, we focused on finding the most consistent methylation changes by selecting only significant CpGs with an absolute Delta *β* > 0.10 when comparing CR-RSV against RW/AS-RSV (**Figure 3A**). This analysis found 28 CpGs (**Table 2**) associated with 23 unique genes; five of them were moderately hypermethylated, the remaining showed a lower methylation level for sequelae cases.

**Table 2:** The 28 most significant DMPs in the comparison RSV sequelae patients vs. CR-RSV cases (FDR *P-*value < 0.01, absolute average *β*-value > 0.10).

CpG_ID	Chr	Position	Location	GN	GG	*P*-Value	Delta *β*
cg21226224	8	55370171	IS	*SOX17*	TSS1500	3.29×10^-04^	***–*** 0.144
**cg24509398**	**1**	**28416532**	**SS**	***EYA3***	**TSS1500**	**2.77×10^-10^**	***–*0.136**
cg11702503	19	6215254	IS	*MLLT1*	Body	2.43×10^-03^	***–*** 0.118
**cg00044440**	**5**	**138671449**	**OS**	***–***	***–***	**6.77×10^-06^**	***–*0.118**
**cg15619333**	**12**	**133759653**	**SS**	***ZNF268***	**5’UTR; Body**	**4.39×10^-09^**	***–*0.116**
cg05800416	8	19460097	IS	*CSGALNACT1*	TSS200; Body; 5’UTR	3.00×10^-04^	***–*** 0.116
**cg12216772**	**10**	**46164062**	**NE**	***ANUBL1***	**5’UTR**	**3.33×10^-08^**	***–*0.115**
**cg06579481**	**7**	**104621597**	**NE**	***–***	***–***	**8.57×10^-06^**	***–*0.112**
cg19977004	11	1482563	IS	*BRSK2*	3’UTR	4.45×10^-04^	***–*** 0.111
cg05116443	20	62562680	IS	*DNAJC5*	Body	1.25×10^-04^	***–*** 0.110
cg08938155	5	77043612	OS	*TBCA*	Body	3.68×10^-03^	***–*** 0.110
**cg15822108**	**4**	**140306311**	**OS**	***NAA15***	**Body**	**4.28×10^-07^**	***–*0.109**
cg19841649	5	4866322	IS	***–***	***–***	7.58×10^-03^	***–*** 0.108
cg26900509	11	127514	IS	*LOC100133161*	Body	3.13×10^-04^	***–*** 0.108
cg13165070	11	2154113	IS	*INS-IGF2; IGF2*	Body; 3’UTR	1.87×10^-03^	***–*** 0.107
cg02077481	16	33939020	IS			9.62×10^-03^	***–*** 0.106
**cg02737727**	**14**	**105561470**	**SS**	***LOC102723354***	**Body**	**1.84×10^-09^**	***–*0.105**
**cg14819618**	**8**	**8180214**	**SE**	***PRAGMIN***	**Body**	**2.26×10^-06^**	***–*0.104**
**cg23621438**	**22**	**25850271**	**OS**	***MIR6817; CRYBB2P1***	**TSS1500; Body**	**1.84×10^-09^**	***–*0.102**
cg05992347	16	33964783	IS	*MIR1826*	TSS1500	4.54×10^-04^	***–*** 0.101
cg16003687	6	168613889	SS	***–***	***–***	3.28×10^-05^	***–*** 0.101
cg04479860	4	190767364	IS	***–***	***–***	7.15×10^-03^	***–*** 0.101
cg06583549	19	46387962	IS	*IRF2BP1*	1^st^ Exon	4.31×10^-04^	***–*** 0.101
cg27552418	4	97598786	OS	***–***	***–***	1.08×10^-03^	0.104
cg25338438	5	161276341	OS	*GABRA1*	5’UTR; TSS1500	1.04×10^-03^	0.107
cg09728337	10	32668256	OS	*EPC1*	TSS1500	5.80×10^-03^	0.110
cg20967739	1	50895827	SE	***–***	***–***	9.25×10^-04^	0.114
cg06259441	3	23957589	NS	RPL15; NKIRAS1	TSS1500; 5’UTR	1.48×10^-03^	0.115

The positions are ordered according to their Delta β-value. The first 23 CpGs exhibit a lower methylation level in CR-RSV than in sequelae cases, while the following 5 DMPs have an opposite pattern. In bold, positions with the lowest P-value, represented in **Figures 5**, **6**. Chr, chromosome; SE, S Shelf; NE, N Shelf; NS, N Shore; SS, S Shore; IS, island; OS, OpenSea; GN, Gene name; GN; GG, Gene group; P-value, FDR P-value.

### Methylation Signature for RSV Sequelae

A total of 9 out of the 28 significant positions with absolute Delta *β* > 0.10 showed an FDR *P*-value < 1×10^-4^, and were selected to evaluate their ability, in terms of methylation levels, to discriminate RW/AS-RSV from CR-RSV groups (**Figure 3B**). All the selected positions exhibit hypomethylation pattern in RW/AS-RSV patients when compared to the CR-RSV group (**Figure 3C**). Two of the nine positions, namely, the cg00044440 (Chromosome 5; OpenSea; FDR *P*-value = 6.77×10^-6^; Delta *β = -*0.12), and the cg06579481 (Chromosome 7; North-Shelf; FDR *P*-value = 8.57×10^-6^; Delta *β = -*0.12), were not annotated to a gene. The remaining seven DMPs, instead, were associated to eight different genes: cg24509398 (FDR *P*-value = 2.77×10^-10^; Delta *β = –*0.14) falls in the body of *EYA3*; cg15619333 (FDR *P*-value = 4.39×10^-9^; Delta *β = –*0.116×10^-1^) locates in the body of *ZNF268*; cg12216772 (FDR *P*-value = 3.33×10^-8^; Delta *β = –*0.12) is in the 5’UTR of *ANUBL1*; cg15822108 (FDR *P*-value = 4.28×10^-7^; Delta *β = –*0.11) settles in the body of *NAA15*; cg02737727 (FDR *P*-value = 1.84×10^-9^; Delta *β = –*0.11) falls in the body of *LOC102723354*; cg14819618 (FDR *P*-value = 2.26×10^-6^; Delta *β = –*0.10) resides within the body of *PRAGMIN*; and finally, cg23621438 (FDR *P*-value = 1.84×10^-9^; Delta *β = –*0.10), locates within the TSS1500 of *MIR6817* and the body of *CRYBB2P1.*

**Figure 4:**
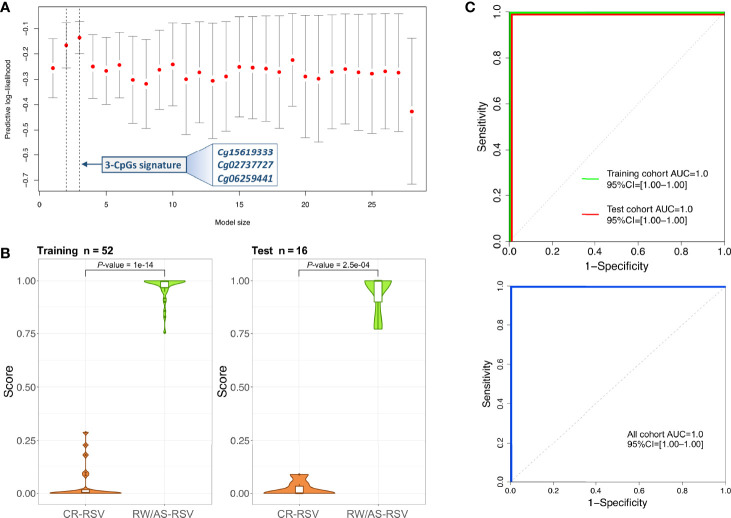
**(A)** Optimal model size according to logistic regression analysis. The x-axis represents the training set predictive log-likelihood, while the y-axis shows the number of genes of the signature. Solid grey bars indicate one standard error (SE) of the predictive log-likelihood. Vertical dashed lines show the model with the best predictive log-likelihood and the model within one-SE of the best model. **(B)** Violin and boxplots of the predicted values from the posterior mean of the optimal model in training and test cohorts. **(C)** ROC curves of the 3-CpGs signature from both training and test cohorts show the area under the curve (AUC) and 95% confidence intervals (CIs). AUC values for the whole cohort are also displayed.

A logistic regression analysis performed with the 28-CpG lead positions found a minimum epigenetic signature composed of three methylation markers (cg15619333, cg02737727, and cg06259441) as the best diagnostic model to differentiate RW/AS-RSV from CR-RSV in the training set (*n* = 52) (**Figure 4A**). The cg15619333 and the cg02737727 were previously described among the candidate CpGs with the lowest FDR *P*-value, while the North Shore cg06259441 resides within two overlapping genes *RPL15* and *NKIRAS1* in chromosome 3. Interestingly, this last position showed an opposite pattern of methylation, being highly methylated in RW/AS-RSV cases.

This 3-position signature showed high diagnostic performance with the training set (*P*-value = 1.00×10^-14^; **Figure 4B**), with AUC value of 100% (95% CI: 1-1) (**Figure 4C**). When the 3-signature model was applied to the test set (*n* = 16), we obtained similar prediction results (*P*-value = 2.50×10^-4^) with an AUC value of 100% (95% CI: 1–1) as in the training set (**Figure 4C**). The diagnosis accuracy of the model was not affected when all samples were analysed together (**Figure 4C**).

### Enrichment Pathways Analysis

**Figure 5:**
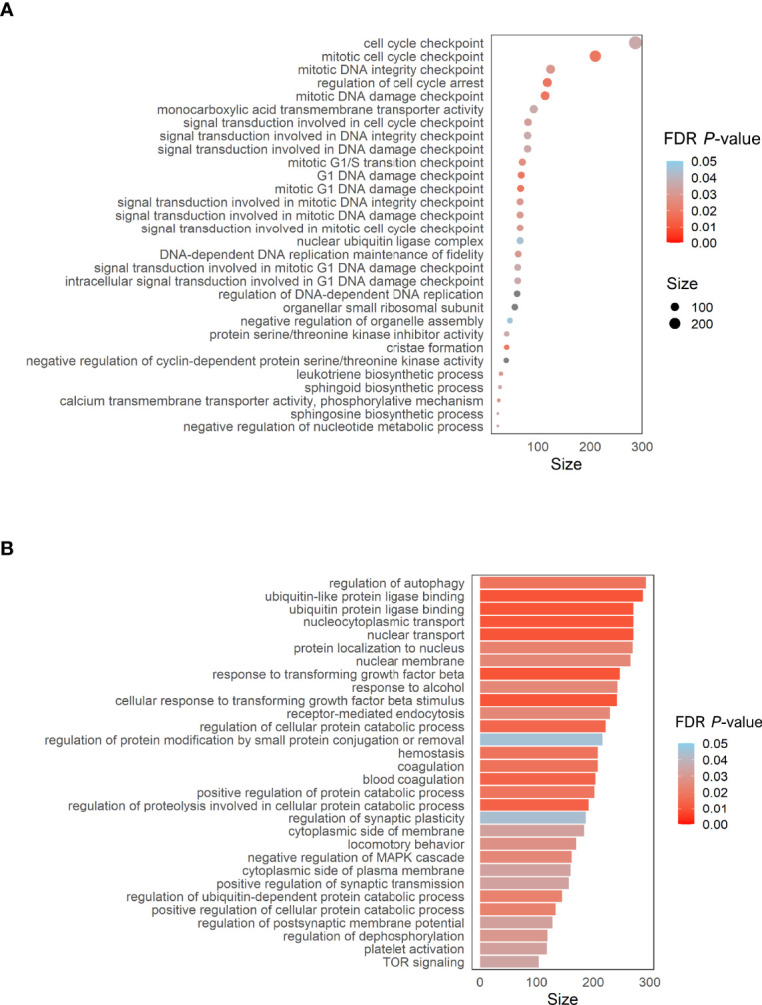
**(A)** Dot plot of the top GO pathways (FDR *P*-value < 0.05) obtained between RW/AS-RSV and CR-RSV (methylglm approach). **(B)** Bar plot of the enrichment pathways analysis performed considering only CpGs within the promoter regions (methylRRA approach). Size along the x-axis indicates the number of genes involved in each pathway. Colors correspond to the different FDR *P*-values associated with the pathways.

To address the potential biological significance of DMPs, a gene set enrichment and pathways analysis was carried out. When comparing the sequelae group against CR-RSV cases, many significantly enriched pathways (FDR *P*-value < 0.05) in the GO database was detected (**Figure 5A**). Among the top overrepresented categories, there were those related to several cell cycle processes, such as the cell cycle checkpoint (FDR *P*-value = 4.71×10^-2^), DNA damage (FDR *P*-value = 2.53×10^-2^), and DNA integrity checkpoint (FDR *P*-value = 2.56×10^-2^). The enrichment pathways analysis performed exclusively on positions overlapping promoters (TSS200 and TSS1500), revealed significant enrichment in the GO ubiquitin ligase binding (FDR *P*-value = 1.21×10^-2^), and response to transforming growth factor beta (FDR p.value =1.21x10^-2^), among other pathways (**Figure 5B**).

### Co-Methylation Modules

We detected 99 co-methylation modules (**Supplementary Figure 1B**) when comparing methylation patterns of RW/AS-RSV against CR-RSV patients. The top one (showing the highest correlation with the RW/AS-RSV phenotype; *P*-value = 1.67×10^-9^; **Supplementary Table 3**; **Supplementary Figure 1C**) includes 3,147 CpGs (**Supplementary Table 3**). This module shows a negative correlation with the trait (–0.65), pointing to a hypomethylation state in RW/AS-RSV concerning the CR-RSV group. This pattern can also be inferred from the heatmap and samples eigengenes plots, with overall higher methylation values in the CR-RSV cohort (**Supplementary Figures 2A, B**). A highly significant correlation between gene significance (GS) and module membership (MM) was observed for this module, meaning that CpGs strongly associated with RW/AS-RSV were also core elements of the module (**Supplementary Figure 2C**). Interestingly, the vast majority of the main hub CpGs within this module corresponds with the previously identified 5.097 DMPs (~92%). Three of them are among the 28-CpGs previously described as most remarkable DMPs between sequelae and non-sequelae groups (cg24509398 [*EYA3*], cg15822108 [*NAA15*], cg15619333 [*ZNF268*]; **Supplementary Table 3**). Genes linked to the most significant module positions were mainly involved in pathways related to GTPase activity, epigenetic modifications, and cell cycle; and molecular functions related to actin, cadherin, and calmodulin binding and also, with focal adhesion components (**Supplementary Table 3**).

### Identification of DMPs Associated With Sequelae Sub-Phenotypes

We next investigated RW-RSV and AS-RSV separately using the same linear model and thresholds described above for the merged sequelae group. When comparing RW-RSV vs. CR-RSV we detected 3,432 significant DMPs, of which 2,663 were hypermethylated, and 769 were hypomethylated in CR-RSV cases. Likewise, the comparison of AS-RSV patients vs. CR-RSV allowed us to identify 1,358 DMPs, with the vast majority being hypermethylated (*n* = 1,059), and 299 being hypomethylated in CR-RSV. A total of 512 significant DMPs were shared by the two contrasts, and also overlap with the most significant DMPs found when comparing respiratory sequelae cases vs. CR-RSV.

**Figure 6:**
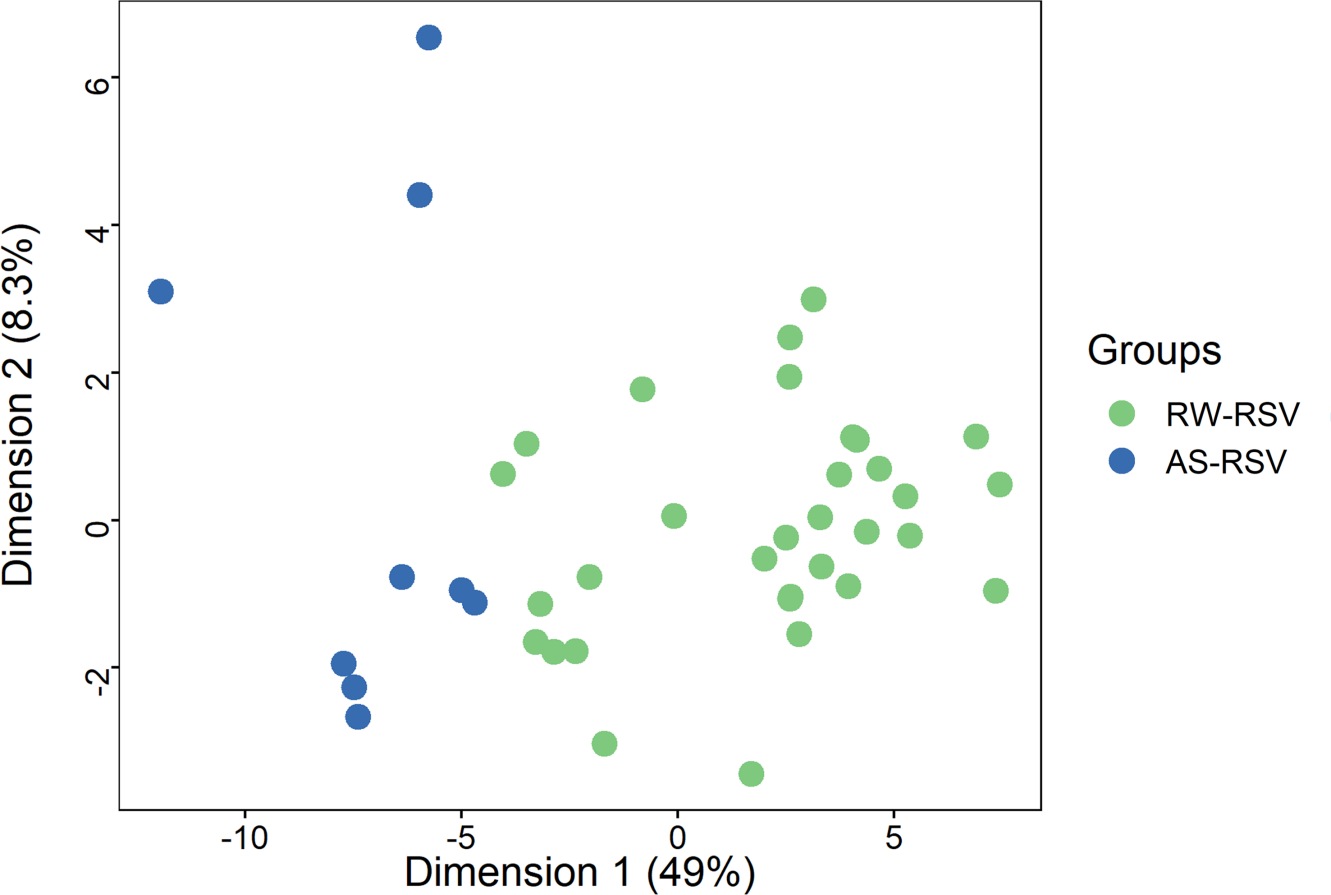
PCA of the most different DMPs (FDR *P*-value < 0.05) shows an almost clear separation between RW-RSV and AS-RSV group.

As shown above, a PCA considering DMPs between sequelae cases vs. CR-RSV patients allows also to separate the two sequelae phenotypic groups in its second component (**Figure 2**: see PC2; accounting for ~9% of the variation). By running a linear model with the contrast AS-RSV vs. RW-RSV and using a less stringent threshold (FDR *P*-value <0.05), a total of 47 significantly DMPs associated with 41 genes could be detected. A PCA was carried out exclusively with these 41 DMPs and the two sequelae phenotypes clearly separate the two groups in its PC1 (49% of the full variance) (**Figure 6**). Of the significant CpGs, 30 positions were found to be hypermethylated in RW-RSV when compared to AS-RSV, while the remaining 17 exhibit the opposite pattern (**Table 3**). The position with the highest difference in *β* value (FDR *P*-value = 1.8×10^-2^; Delta β >0.14) was the cg18873878 located within the *TP73* gene, while the most significant CpGs (FDR *P*-value = 0.11×10^-1^) was the cg05838113 located within the body of *ADAM8* gene.

**Table 3:** PDMPs between AS-RSV and RW-RSV groups are ordered by Delta *β*-values (FDR *P-*value < 0.05).

CpG_ID	Chr	Position	Location	GN	GG	*P*-value	Delta *β*
**cg18873878**	**1**	**3607116**	**IS**	***TP73***	**Body; TSS200**	**0.018**	**0.141**
cg19332572	11	65321591	IS	*LTBP3*	Body	0.011	0.118
cg07367519	22	40075288	IS	*CACNA1I*	Body	0.041	0.114
cg11507793	6	29856363	IS	*HLA-H*	Body	0.041	0.113
cg10274606	14	73118334	OS			0.041	0.109
cg14065121	9	77643271	IS	*C9orf41*	1^st^Exon; 5’UTR	0.011	0.106
cg27594116	9	100069897	IS	*CCDC180; C9ORF174*	TSS200; Body	0.031	0.105
cg22371961	1	169132356	OS	*NME7*	Body	0.041	0.091
**cg05838113**	**10**	**135082349**	**IS**	***ADAM8***	**Body**	**0.010**	**0.086**
cg02478023	19	57351322	IS	*MIMT1; PEG3; ZIM2;*	TSS1500; 5’UTR	0.036	0.082
cg25612428	5	10649867	IS	*ANKRD33B*	Body	0.040	0.082
cg25550913	13	114783665	IS	*RASA3*	Body	0.042	0.077
cg05472874	22	44258179	IS	*SULT4A1*	1^st^Exon	0.030	0.075
cg09519644	12	85401946	OS	***–***	***–***	0.011	0.075
cg11946459	6	29911558	SS	*HLA-A*	Body	0.011	0.073
cg01848660	2	68269960	OS	*C1D*	3’UTR	0.041	0.073
cg01979489	16	332603	IS	*ARHGDIG; PDIA2*	Body; TSS1500	0.041	0.072
cg00664920	16	2664747	IS	*LOC652276*	Body	0.021	0.067
cg09046688	9	75621983	OS	***–***	***–***	0.041	0.065
cg01997696	20	43374401	IS	*KCNK15*	TSS200	0.036	0.063
cg14377711	16	1384369	IS	*BAIAP3*	5’UTR; TSS200	0.041	0.060
cg16740746	16	78134345	SS	*WWOX*	Body	0.037	0.059
cg23215256	7	631862	IS	*PRKAR1B*	Body	0.041	0.058
cg01077616	6	42017945	NS	*CCND3; TAF8*	TSS1500	0.022	0.056
cg23861120	12	67835705	OS	***–***	***–***	0.014	0.046
cg14390580	17	35873008	IS	*DUSP14*	3’UTR	0.041	0.042
cg18560442	1	39174410	IS	***–***		0.041	0.040
cg15842722	20	23499644	OS	*CSTT*	TSS200	0.041	0.035
cg25326090	19	47197766	SS	*PRKD2*	Body	0.044	0.033
cg05801818	22	23262424	OS	***–***		0.044	0.024
cg08103551	11	76777993	IS	*CAPN5*	1^st^Exon; 5’UTR	0.041	***–*** 0.007
cg10504753	13	100258763	IS	*CLYBL*	TSS200	0.041	***–*** 0.010
cg06787731	14	38069079	IS	***–***	***–***	0.044	***–*** 0.012
cg08806408	16	51185001	IS	*SALL1*	TSS1500; Body	0.021	***–*** 0.016
cg14181391	20	33265182	IS	*PIGU*	TSS200	0.041	***–*** 0.017
cg09672082	5	271577	IS	*PDCD6*	TSS200	0.036	***–*** 0.018
cg25372335	10	82168065	IS	*C10orf58*	TSS200	0.041	***–*** 0.020
cg21345913	18	61822270	OS	*LOC284294*	Body	0.041	***–*** 0.023
cg07648504	19	21262035	NE	***–***	***–***	0.041	***–*** 0.034
cg18693345	5	2754148	IS	*C5orf38*	Body	0.041	***–*** 0.035

Chr, chromosome; SE, S Shelf; NE, N Shelf; NS, N Shore; SS, S Shore; IS, island; OS, OpenSea; GN, Gene name; GN, GG, Gene group; P-value, FDR P-value.

In bold, positions with higest difference in methylation and lowest P-value, respectively.

## Discussion

It is known that host factors might impact subsequent respiratory morbidity in RSV infection, but the underlying biological mechanisms through which recurrent wheezing and asthma are prone to emerge after RSV infection are still unknown.

**Figure 7:**
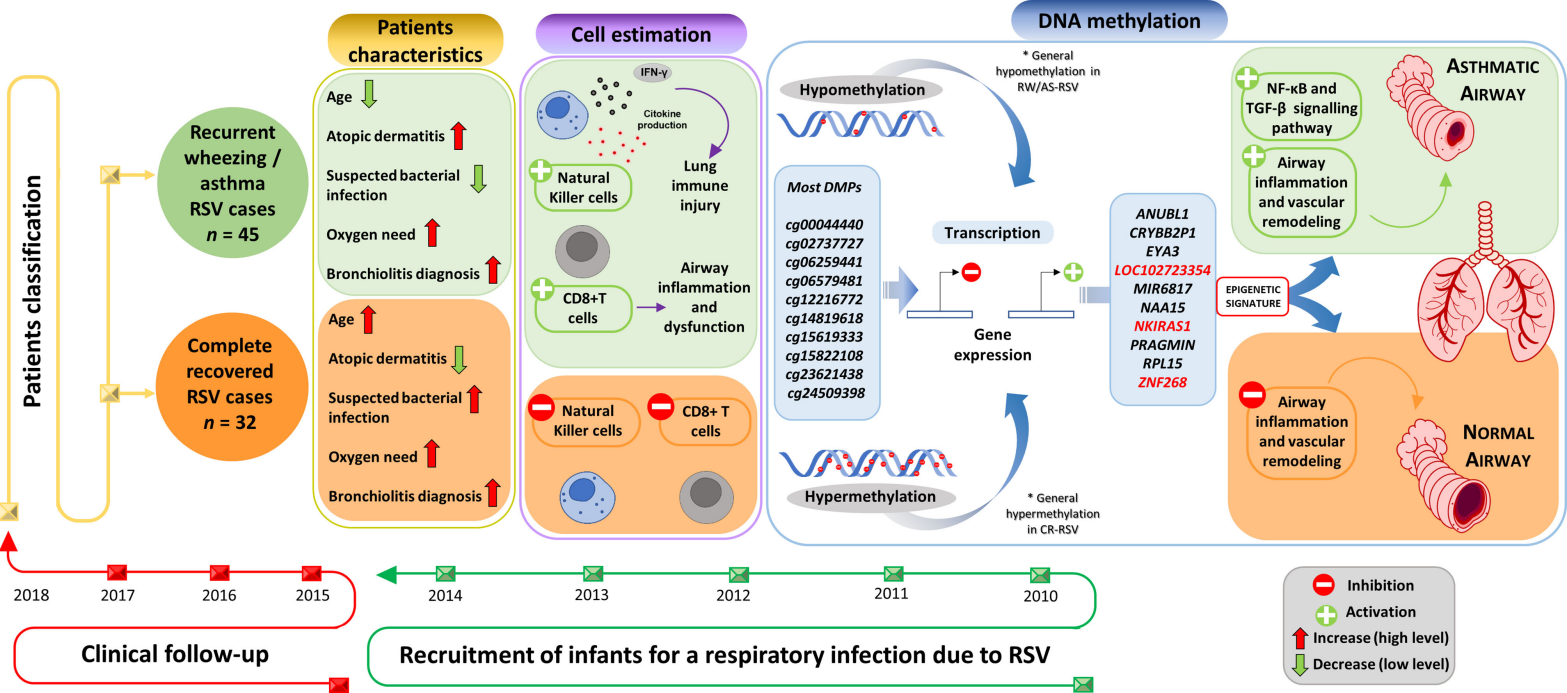
Study design and overview of main results.Supplementary Material Legend.

The analysis of the DNA methylome in RSV infected children allowed us to find >5.000 DMPs mainly located in inflammatory genes when comparing patterns in individuals with sequelae from those completely recovered (**Figure 7**). Overall, the findings suggest that epigenetic mechanisms might play a fundamental role in the long-term sequelae after RSV infection. The association between RSV infection and the subsequent development of wheezing and/or asthma in infants has been widely reported (7, 8, 45, 46). This correlation seems to be very complex owed to the several host environmental factors that influence the expression of respiratory sequelae developed after the infection, such as genetics, age, prematurity, RSV bronchiolitis, atopic dermatitis, and maternal asthma. The patients analyzed in the present study had a very similar clinical course, with the particularity that those developing respiratory sequelae were two months younger than those completely recovered and show more incidence of atopic dermatitis; similar findings were already described to be related with respiratory morbidity after RSV infection (47).

Estimation of the relative proportion of cell types in peripheral blood samples of patients revealed a statistically significantly higher proportion of NK and CD8T cells in children with respiratory sequelae than those with complete recovery (**Figure 7**). This is in line with evidences demonstrating that RSV infection can induce severe acute lung immune injury promoting the accumulation of lung NK cells at the early stage of infection in mice, as a consequence of the increased production of IFN-γ (48). Similar characteristics were described for CD8+ T cells, which are activated after RSV infection to produce inflammatory cytokines (49), and seem to enhance airway inflammation and airway dysfunction in mice (50). However, there is growing body of evidence suggesting that NK cells, as well as CD8+T cells are involved in both the promotion and inhibition of allergic lung inflammation and airway diseases (51).

After correcting the linear model for cell composition, age, and gender, the outcomes of the preliminary analysis revealed 5,097 DMPs when comparing CR-RSV vs. RW/AS-RSV. The most significant CpG (FDR *P*-value = 2.77×10^-10^; Delta β = -0.14) was cg24509398 within the *EYA3* gene, a tyrosine phosphatase involved in DNA repair and in distinguishing apoptotic and repair responses to genotoxicity. Recently, Wang et al. (52) suggested that the over-expression for this gene in patients with pulmonary arterial hypertension (PAH) might stimulate the survival of pulmonary vascular cells in the presence of DNA damage causing vascular remodeling, a typical feature of PAH. Although PAH and asthma are considered two different and unrelated clinical phenomena, there are pieces of evidence demonstrating that they share some pathological features such as inflammation, smooth muscle constriction, and proliferation (53). Although DNA methylation in the CpG selected might not be exerting strong changes in the expression of *EYA3*, the putative involvement of this gene in the vascular remodeling (a key process in the asthma pathology), might indicate a possible connection between the altered methylation status of the gene and its uncontrolled gene expression.

To test the diagnostic potential of epigenetic markers in the context of RSV sequelae, we carried out a logistic regression analysis using the top 28 DMPs. We found a 3- CpGs position model (related to *ZNF2698*, *LOC102723354* and the overlapping *RPL15*/*NKIRAS1* genes; AUC = 1.00) as the best epigenetic signature to distinguish RW/AS-RSV phenotypes from CR-RSV in both training and test sets as well as in the whole cohort (**Figure 7**). While *LOC102723354* is related to an uncharacterized long non-coding RNA (lncRNA), whose function is still completely unknown, *ZNF268* and *NKIRAS1* are known to regulate, at a different level and in a different way, the nuclear factor ‘kappa-light-chain-enhancer’ of activated B-cells (NF-κB) (54, 55), an important transcription factor that plays a critical role in the production of many inflammatory cytokines. NF-κB is involved in activating and coordinating both innate and adaptive immune responses; it is found to be associated with allergic airway diseases, and it is activated in bronchial asthmatic patient biopsies and airway epithelium from mice (56).

Among the pathways found to be significantly enriched from the functional analysis, those involved in the cell cycle checkpoint are the most significantly enriched. These finding might suggest that cell proliferation may play an important role in the pathogenesis of wheezing and asthma after RSV infection. Besides, significant enrichment in pathways involved in DNA damage and integrity checkpoint was also found, probably suggesting that the cellular DNA damage machinery is activated and exploited by viruses which have acquired the ability to manipulate the key regulators of these pathways to promote their own replication. Considering the positions residing within promoters, we identified a significant enrichment for the nuclear ubiquitin ligase complex pathway. It has been shown that allergic airway inflammation associated with rhinovirus infection leads to the upregulation of the E3 ubiquitin ligase midline 1 (*MID1*) in mouse bronchial epithelium, thus, the inhibition of *MID1* attenuates rhinovirus-induced airway inflammation and asthma exacerbations (57). In addition, the transforming growth factor-beta (TGF-β) signaling pathway was also found to be significant (FDR *P*-value = 1.21x10^-2^) in promoters’ enrichment pathways. This cytokine’s family seems to play a critical role in the development of airway inflammation and remodeling in asthma (58, 59). In asthmatic airways, TGF-β can induce an antiapoptotic effect in the airway epithelial cells through the SMAD signaling pathway and can trigger apoptosis activating MAPK signaling pathways (60–62). Significant DMPs fall within genes involved in the TFG-β related pathways (e.g. *BMP2*, *FLCN*, *ING2*, *PMEPA1*, *PRDM16*, *TGFB1I1* and *TGFBR3*), in line with previous studies that reported an association between these genes and the increased asthma risk (63–68). All over, these results would suggest that the regulation of the TGF-β could be involved in the induction of a moderate or severe course of asthma, a common respiratory sequela observed in children after RSV infection.

In addition, we found a set of co-methylated positions that were significant negatively correlated with the sequelae phenotype, pointing to a hypomethylation state of this CpGs block in RW/AS-RSV patients. Genes associated with these positions pointed out to GTPase signaling as the most significant route in sequelae after RSV infection. Other related significant pathways were those involved in Ca^2+^ metabolism, actin binding and cell junctions. The Rho family of GTPases has been proposed as a promising therapeutic target for asthma, and it is known to play an important role in the pathophysiology of asthma, including airway smooth muscle contraction, airway hyper‐responsiveness and bronchial epithelial barrier dysfunction and recently, in mesenchymal stem cell differentiation and migration for airway remodeling and repairing (69).

A moderate number of statistically significant DMPs emerged when analyzing wheezing and asthma phenotypes separately. The highest difference in Delta *β* (0.148) corresponds to the cg18873878 observed within the TSS200 region of the *TP73* gene being hypermethylated in RW-RSV children in comparison with those with asthma. *TP73* gene encodes a member of the p53 family of transcription factors involved in cellular responses to stress and development; its role in airway epithelium is unknown, even if its homolog, *TP63*, is found to be essential for tracheobronchial epithelium development and homeostasis (70).Another interesting position with a lower methylation pattern in children with asthma was the cg05838113 located within the body of the *ADAM8* gene, an ADAM Metallopeptidase Domain which is linked to asthma. It seems that mice with allergic airway inflammation (AAI) show higher levels of expression of *ADAM8* in airway epithelium and airway inflammatory cells (71). In addition, increased expression of *ADAM8* was observed in the sputum and endobronchial biopsies of patients with moderate and severe asthma (72).

There are a few limitations in the present study. First, the main limitation is the limited number of patients analyzed, and the relative unbalanced in the available samples for the different RSV phenotypes studied. This fact led us to consider a less conservative threshold for the contrast analysis RW-RSV vs. AS-RSV which, in consequence, might increase the probability for false positives. In turns, our study is better powered for the recurrent wheezing outcome. Second, the clinical history of patients is incomplete for some co-variates of interest. Third, the three years of follow up could be not sufficient to discriminate between recurrent wheezing and asthma, being patients with recurrent wheezing at risk to develop asthma in the following years; however, a three-year follow-up should highly capture the diagnosis of asthma in those children with predisposition to develop the disease. Finally, in our cohort, there is higher bacterial superinfection rates in CR-RSV (71.9%) vs. the RSV sequelae cohort (28.9%). The presence of bacteria could have impacted the epigenetics results if the changes observed were due to bacterial presence rather than differences in response to the viral infection. However, it is important to note that bacterial superinfection, as defined in our study, was not always based on a bacterial isolation in a sterile site, but rather frequently based on suggestive clinical symptoms, radiological findings and/or analytical values such as elevated biomarkers (i.e procalcitonin).To account for the mentioned limitations, statistical analyses were carried out considering all possible confounding factors. Even so, further studies will be needed to corroborate the main findings of the present study, as well as other complementary epigenetic studies that takes into account other epigenomic modifications e.g. in the chromatin.

## Conclusion

In conclusion, there is suggestive evidence in the present study indicating that epigenetic factors might contribute to the susceptibility to develop recurrent wheezing and asthma after RSV infection. Many DMPs associated with respiratory sequelae developed after RSV infection were detected, with remarkable patterns of methylation profiles observed for genes involved in airway inflammation processes. Functional analysis considering all significant CpGs between sequelae and complete recovery patients revealed significant enrichment for pathways involved in cell cycle checkpoint, DNA damage and integrity checkpoint. In addition, we reported a 3-CpGs epigenetic signature that might be of interest as a diagnostic tool for RSV sequelae. DNA methylation might play a fundamental role in the development of asthma and/or wheezing after RSV infection and could explain the different post-infection sequelae observed. Further investigation using larger cohorts, as well as transcriptomic studies, would be needed to further disentangle the role of epigenomics in asthma and other clinical manifestations (e.g. wheezing), as well as to validate epigenetic biomarkers that allow predicting with precision the different respiratory sequelae that can emerge after infection.

## Data Availability Statement

The data presented in the study are deposited on Gene Expression Omnibus (GEO) repository, Accession number: GSE199334.

## Ethics Statement

The studies involving human participants were reviewed and approved by the Ethics Committee of Clinical Investigation of Galicia (CEIC 2010/015; updated version 2017/07/26, reg 2017/398). Written informed consent to participate in this study was provided by the participants’ legal guardian/next of kin.

## Author Contributions

FM-T, AS, and JG conceived and designed the experiments. IR-C, MC-L and MTG-L were involved in sample recruitment and collection of clinical data. SP, AC, AD-L performed the experiments. SP, and AG-C analyzed the data. SP, AG-C, and AS wrote the first draft of the manuscript, and was contributed by FM-T. All authors read and approved the final manuscript.

## Funding

This study received support from Instituto de Salud Carlos III (ISCIII): GePEM (PI16/01478/Cofinanciado FEDER; AS), DIAVIR (DTS19/00049/Cofinanciado FEDER, AS), Resvi-Omics (PI19/01039/Cofinanciado FEDER, AS), Agencia Gallega de Innovación (GAIN): Grupos con Potencial de Crecimiento (IN607B 2020/08, AS); Agencia Gallega para la Gestión del Conocimiento en Salud (ACIS): BI-BACVIR (PRIS-3, AS), and CovidPhy (SA 304 C, AS); ReSVinext (PI16/01569/Cofinanciado FEDER, FM-T), Enterogen (PI19/01090/Cofinanciado FEDER, FM-T) and consorcio Centro de Investigación Biomédica en Red de Enfermedades Respiratorias (CB21/06/00103; FM-T; GEN-COVID (IN845D 2020/23, FM-T) and Grupos de Referencia Competitiva (IIN607A2021/05, FM-T). AD-L is funded by a contract “Juan Rodés” from ISCIII (JR17/00016). ABC is a Miguel Servet researcher (ISCIII; CP17/0008). The funders were not involved in the study design, collection, analysis, interpretation of data, the writing of this article or the decision to submit it for publication.

## Conflict of Interest

IR-C has received honoraria from GSK, Pfizer, Sanofi Pasteur and MSD for taking part in advisory boards and expert meetings and for acting as a speaker in congresses outside the scope of the submitted work. IR-C has also acted as subinvestigator in randomized controlled trials of Ablynx, Abbot, Seqirus, Sanofi Pasteur MSD, Sanofi Pasteur, Cubist, Wyeth, Merck, Pfizer, Roche, Regeneron, Jansen, Medimmune, Novavax, Novartis and GSK. FM-T has received honoraria from GSK group of companies, Pfizer Inc, Sanofi Pasteur, MSD, Seqirus, Biofabri and Janssen for taking part in advisory boards and expert meetings and for acting as a speaker in congresses outside the scope of the submitted work. FM-T has also acted as principal investigator in randomized controlled trials of the above-mentioned companies as well as Ablynx, Gilead, Regeneron, Roche, Abbott, Novavax, and MedImmune, with honoraria paid to his institution.

The remaining authors declare that the research was conducted in the absence of any commercial or financial relationships that could be construed as a potential conflict of interest.

## Publisher’s Note

All claims expressed in this article are solely those of the authors and do not necessarily represent those of their affiliated organizations, or those of the publisher, the editors and the reviewers. Any product that may be evaluated in this article, or claim that may be made by its manufacturer, is not guaranteed or endorsed by the publisher.

## Supplementary Material

Supplementary Figure 1
**(A)** Selection of the soft-thresholding power. Plots showed a correlation between soft-thresholding powers and both the scale-free fit index (upper) and the mean connectivity (lower). **(B)** Clustering dendrogram of CpGs and co-methylation modules detected represented by different colors. **(C)** Average significance of all CpGs in each module.

Supplementary Figure 2
**(A)** *β*-values heatmap of the CpGs from the pink module. Samples eigengenes values are also represented **(B)** Differences in samples eigengenes values from pink module between RW/AS-RSV and CR-RSV groups. **(C)** Correlation between gene significance (GS) for sequelae phenotype and module membership (MM) in the pink module. **(D)** Over-representation analysis of gene ontology terms using genes in the pink module (BP, biological processes; MF, molecular functions; CC, cellular components).
