# Supplementary figures and images for: Role and Diagnostic Performance of Host Epigenome in Respiratory Morbidity after RSV Infection: The EPIRESVi Study
Source: Front Immunol. 2022 May 10;13:875691. doi: 10.3389/fimmu.2022.875691 (PMC9128527; doi:10.3389/fimmu.2022.875691)

**A**

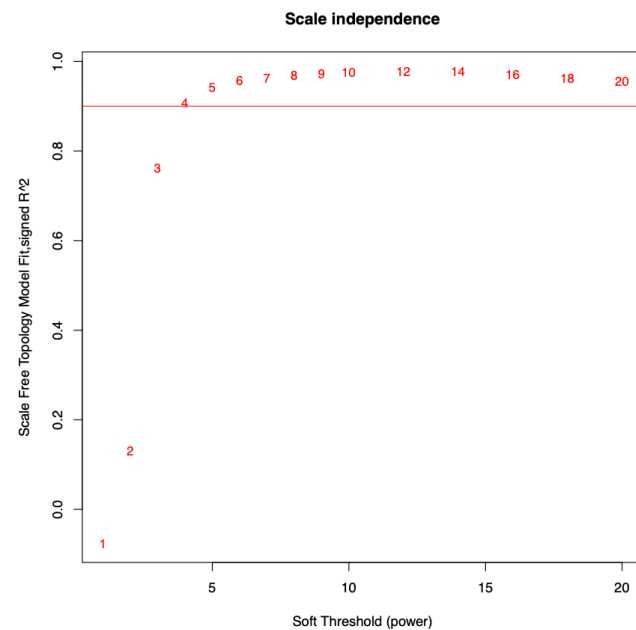

**B**

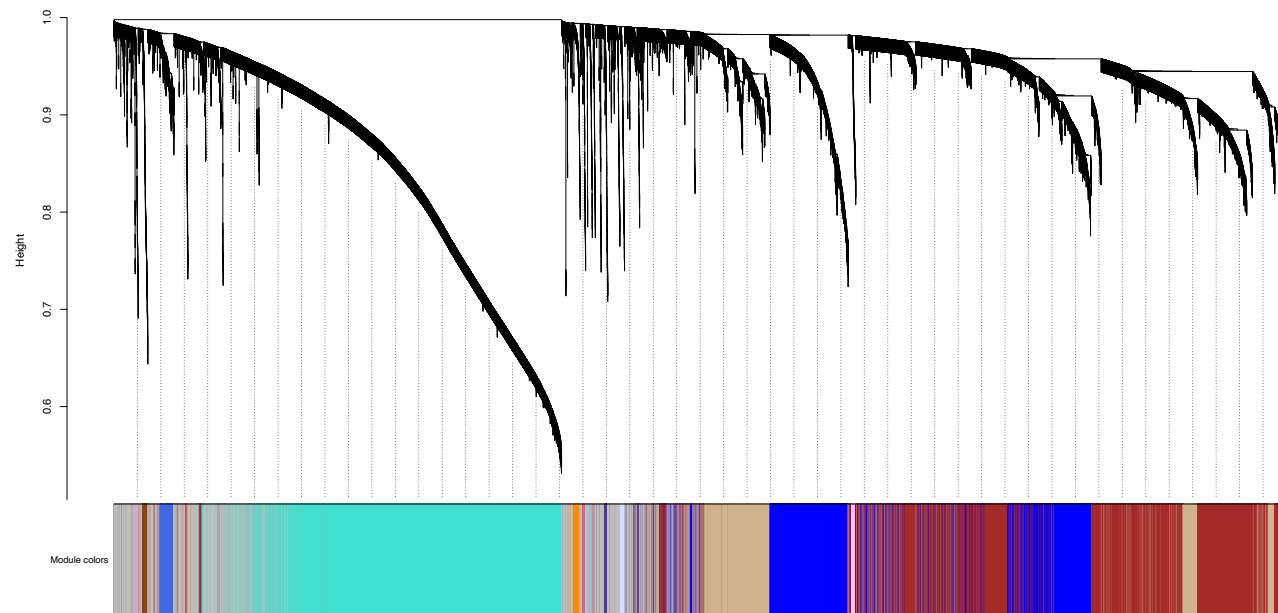

**C**

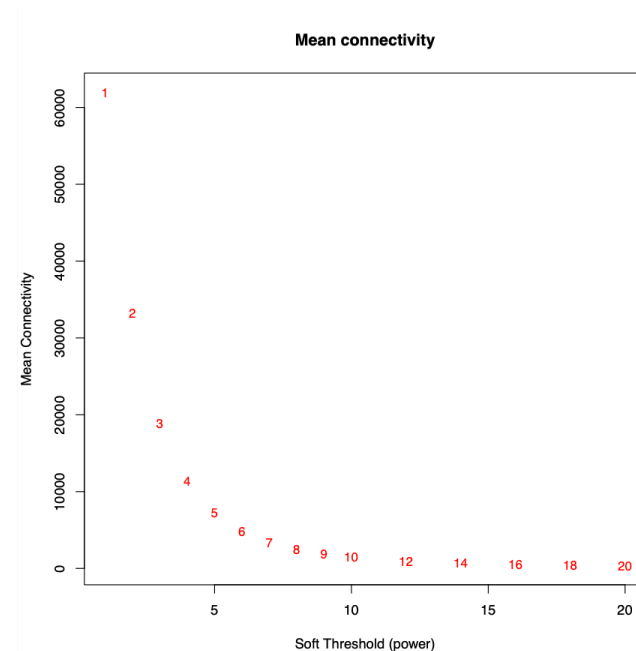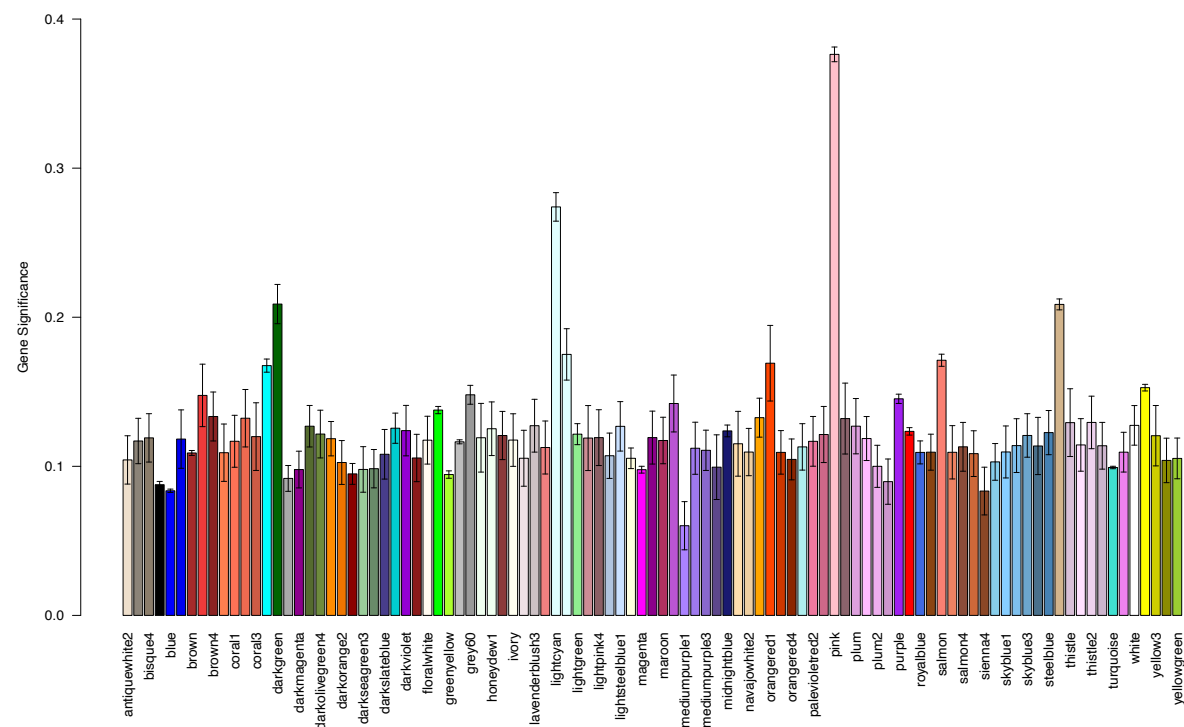

Supplement: Supplementary Figure 1 — (A) Selection of the soft-thresholding power. Plots showed a correlation between soft-thresholding powers and both the scale-free fit index (upper) and the mean connectivity (lower). (B) Clustering dendrogram of CpGs and co-methylation modules detected represented by different colors. (C) Average significance of all CpGs in each module. [file Image_1.pdf]

A

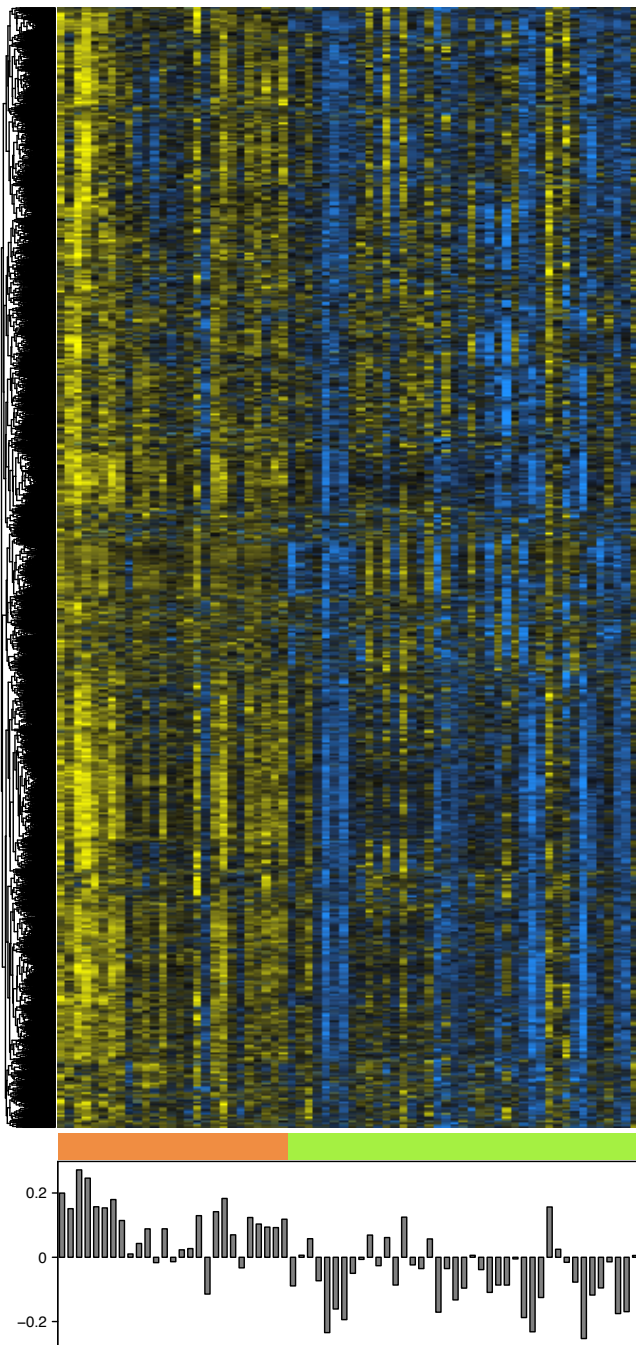

B

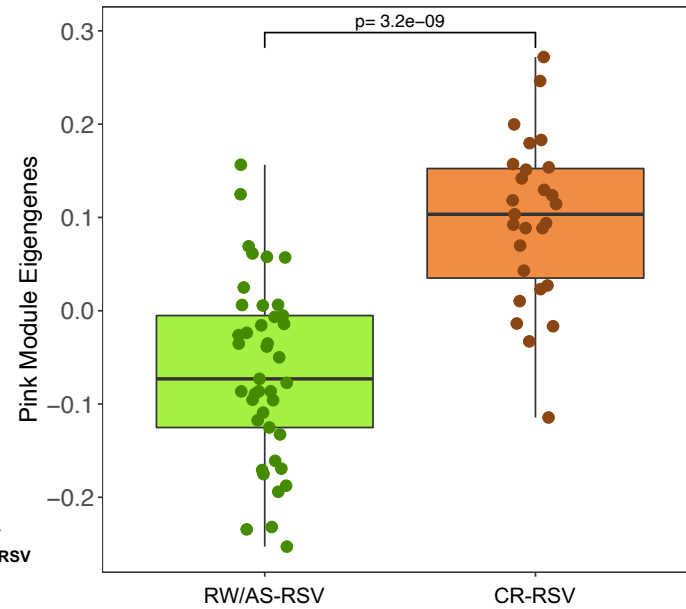

C

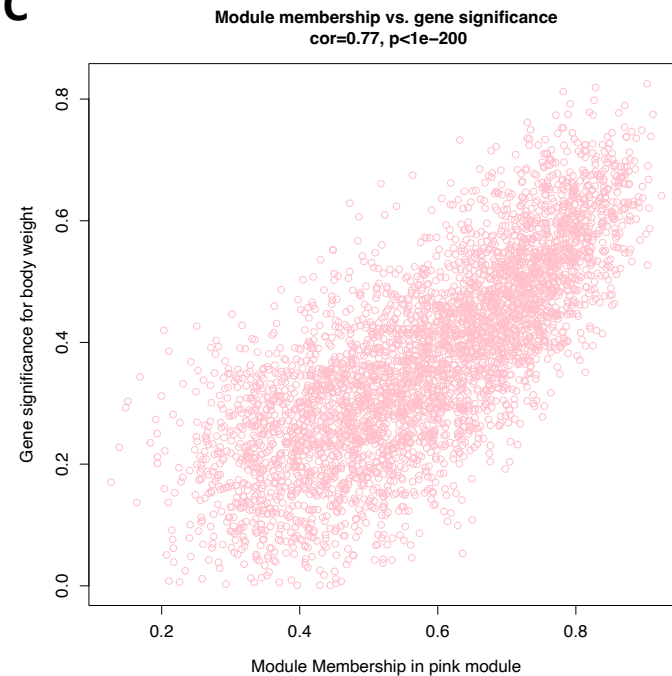

D

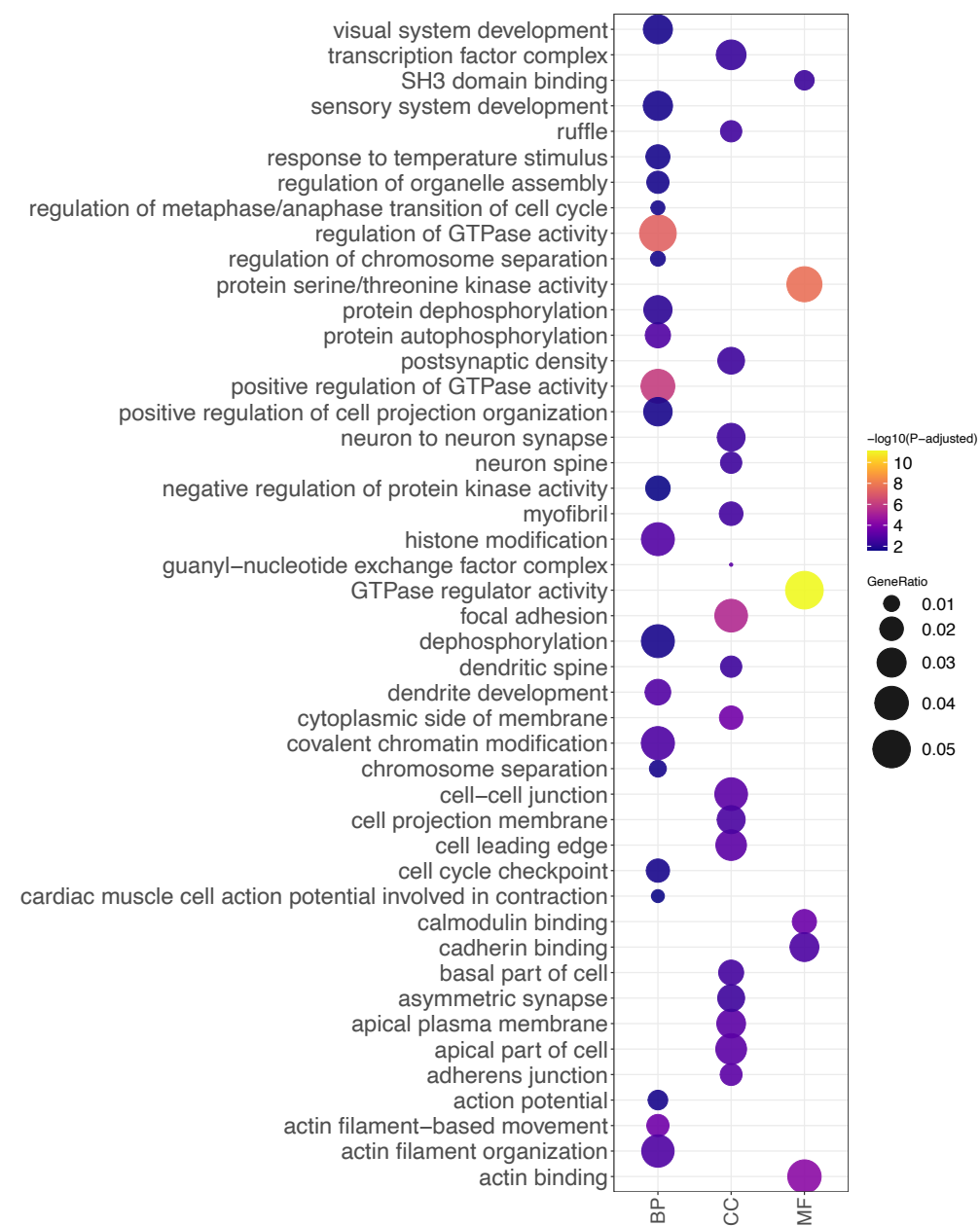

Supplement: Supplementary Figure 2 — (A) β-values heatmap of the CpGs from the pink module. Samples eigengenes values are also represented (B) Differences in samples eigengenes values from pink module between RW/AS-RSV and CR-RSV groups. (C) Correlation between gene significance (GS) for sequelae phenotype and module membership (MM) in the pink module. (D) Over-representation analysis of gene ontology terms using genes in the pink module (BP, biological processes; MF, molecular functions; CC, cellular components). [file Image_2.pdf]
