# Supplementary material for: Role and Diagnostic Performance of Host Epigenome in Respiratory Morbidity after RSV Infection: The EPIRESVi Study
Source: Front Immunol. 2022 May 10;13:875691. doi: 10.3389/fimmu.2022.875691 (PMC9128527; doi:10.3389/fimmu.2022.875691)
Supplement: Supplementary file 3 [file DataSheet_1.docx]

**ANNEX I: Listo of the study researchers of GENDRES network**

Miriam Cebey López, Antonio Salas Ellacuriaga, Ana Vega Gliemmo, José Peña Guitián, Alexa Regueiro, Antonio Justicia Grande, Leticia Pías Peleteiro, María López Sousa, María Jose de Castro, Carmen Curros Novo, Elena Rodrigo, Miriam Puente Puig, Rosaura Leis Trabazo, Nazareth Martinón Torres, Alberto Gómez Carballa, Jacobo Pardo Seco, Antonio Justicia Grande, José María Martinón Sánchez, Belén Mosquera Pérez, Isabel Villanueva González, Lorenzo Redondo Collazo, Carmen Rodríguez-Tenreiro, María del Sol Porto Silva and Federico Martinón Torres (Área Asistencial Integrada de Pediatría and GENVIP, Hospital Clínico Universitario, Santiago de Compostela); Máximo Francisco Fraga Rodríguez, Orlando Fernández Lago, José Ramón Antúnez (Biobank, Servicio Anatomía Patológica, Hospital Clínico Universitario, Santiago de Compostela); Enrique Bernaola Iturbe, Laura Moreno Galarraga, Jorge Álvarez, Mercedes Herranz, Francisco Gil, Eva Gembero, Jorge Rodríguez (Hospital Materno Infantil Virgen del Camino, Pamplona); Teresa González López, Delfina Suarez Vázquez, Ángela Vázquez Vázquez, Susana Rey García, Nathalie Carreira Sande, Ana López Fernández, Nuria Romero Pérez (Complejo Hospitalario Universitario de Orense); José Antonio Couceiro Gianzo, Nazareth Fuentes Perez (Complejo Hospitalario Universitario de Pontevedra); Francisco Giménez Sánchez, Miguel Sánchez Forte (Hospital Torrecárdenas, Almería); Cristina Calvo Rey, María Luz García García, Iciar Olabarrieta Arnal, Adelaida Fernández Rincón (Hospital Severo Ochoa de Madrid); Ignacio Oulego Erroz, David Naranjo Vivas, Santiago Lapeña, Paula Alonso Quintela, Jorge Martínez Sáenz de Jubera, Estibaliz Garrido García (Hospital de León); Cristina Calvo Monge, Eider Oñate Vergara (Hospital de Donostia, San Sebastián); Jesús de la Cruz Moreno, Mª Carmen Martínez Padilla, Eugenia Villanueva Martínez, Ana González Espín, María de las Mercedes Martínez Rebollo, María Rocío Martín Moya (Complejo Hospitalario de Jaén); Manuel Baca Cots (Hospital Quirón, Málaga), David Moreno Pérez, Ana Cordón Martínez, Antonio Urda Cardona, José Miguel Ramos Fernández, Esmeralda Núñez Cuadros (Hospital Carlos Haya, Málaga); Susana Beatriz Reyes, María Cruz León León, Santiago Alfayate (Hospital Virgen de la Arrixaca, Murcia); Cristina Calvo, Carlos Grasa, Cristian Quintana Ortega, Leticia La Banda Montalvo, María Lopez Cerdán, Ana Dominguez Castells (Hospital Universitario La Paz, Madrid); Francisco Giménez Sánchez (Hospital Inmaculada); Andrés J. Alcaraz Romero (Hospital Universitario de Getafe); Roi Piñeiro (Hospital General de Villalba); Juan Ignacio Sánchez Díaz, Alba Palacios (Hospital 12 Octubre); Elvira González Salas, Sira Fernández De Miguel (Hospital Clínico de Salamanca); Belen Joyanes Abancens, Esther Aleo Lujan (Hospital clínico San Carlos); Alfredo Tagarro García, María Luisa Herreros, Rut del Valle, Libertad Latorre Navarro (Hospital Universitario Infanta Sofía); María Concepción Zazo Sanchidrián, Mariano Esteban, Marta González Lorenzo, Mª Carmen Vicent Castello (Hospital General Universitario de Alicante); Lorena Moreno Requena, Juan Luis Santos Pérez (Complejo Hospitalario Universitario de Granada); César Gavilán Martín, Lucía González-Moro Azorín (Hospital Universitario de San Juan de Alicante); Monterrat López Franco, Manuel Silveira Cancela (Hospital de Burela); María José Cilleruelo Ortega, Luz Golmayo, Francisca Portero Azorín (Hospital Universitario Puerta de Hierro-Majadahonda); Andrés Concha Torre, Lucía Rodríguez García (Hospital Universitario Central de Asturias); Carlos Rodrigo Gonzalo de Liria, Andrés Antón Pagarolas (Hospital Vall d'Hebron); Maria Méndez, Critina Prat (Hospital Germans Trias i Pujol); Jesús López-Herce, Miriam García Samprudencio, Gema Manrique Martín, Paula García Casas (Hospital General Universitario Gregorio Marañón); María Jesús Cabero (Hospital Valdecilla); Miguel Lillo Lillo, Marta Pareja (Hospital General de Albacete); Pablo Rojo, Cristina Epalza (Hospital 12 Octubre); Adriana Navas Carretero, Estefanía Barral, Miriam Herrera (Hospital Infanta Leonor); Elvira Cobo Vazquez (Hospital Universitario Fundación de Alcorcón); Elena del Castillo Navío (Hospital Materno Infantil de Badjoz); Patricia Flores Perez (Hospital del Niño Jesús); Paula García Casas (Hospital Ramón y Cajal); Javier Pilar Orive, Elva Rodriguez Merino (Hospital Univeristario Cruces); Ana Pérez Aragón (HMI Virgen de las Nieves de Granada); Mª Yolanda Ruiz del Prado (Hospital San Pedro); David Moreno, Beatriz Carazo (Hospital Carlos Haya); Jordi Antón (Hospital Sant Joan de Déu). Further details may be consulted at http://www.gendres.org or www.genvip.eu
